# Subcellular positioning during cell division and cell plate formation in maize

**DOI:** 10.3389/fpls.2023.1204889

**Published:** 2023-07-07

**Authors:** Lindy A. Allsman, Marschal A. Bellinger, Vivian Huang, Matthew Duong, Alondra Contreras, Andrea N. Romero, Benjamin Verboonen, Sukhmani Sidhu, Xiaoguo Zhang, Holly Steinkraus, Aimee N. Uyehara, Stephanie E. Martinez, Rosalie M. Sinclair, Gabriela Salazar Soriano, Beatrice Diep, Dawson Byrd V., Alexander Noriega, Georgia Drakakaki, Anne W. Sylvester, Carolyn G. Rasmussen

**Affiliations:** ^1^ Department of Botany and Plant Sciences, Center for Plant Cell Biology, University of California, Riverside, Riverside, CA, United States; ^2^ Department of Molecular Biology, University of Wyoming, Laramie, WY, United States; ^3^ Department of Plant Sciences, University of California, Davis, Davis, CA, United States

**Keywords:** mitosis, maize, cell plate, phragmoplast, microtubule

## Abstract

**Introduction:**

During proliferative plant cell division, the new cell wall, called the cell plate, is first built in the middle of the cell and then expands outward to complete cytokinesis. This dynamic process requires coordinated movement and arrangement of the cytoskeleton and organelles.

**Methods:**

Here we use live-cell markers to track the dynamic reorganization of microtubules, nuclei, endoplasmic reticulum, and endomembrane compartments during division and the formation of the cell plate in maize leaf epidermal cells.

**Results:**

The microtubule plus-end localized protein END BINDING1 (EB1) highlighted increasing microtubule dynamicity during mitosis to support rapid changes in microtubule structures. The localization of the cell-plate specific syntaxin KNOLLE, several RAB-GTPases, as well as two plasma membrane localized proteins was assessed after treatment with the cytokinesis-specific callose-deposition inhibitor Endosidin7 (ES7) and the microtubule-disrupting herbicide chlorpropham (CIPC). While ES7 caused cell plate defects in *Arabidopsis thaliana*, it did not alter callose accumulation, or disrupt cell plate formation in maize. In contrast, CIPC treatment of maize epidermal cells occasionally produced irregular cell plates that split or fragmented, but did not otherwise disrupt the accumulation of cell-plate localized proteins.

**Discussion:**

Together, these markers provide a robust suite of tools to examine subcellular trafficking and organellar organization during mitosis and cell plate formation in maize.

## Introduction

Division is an essential step in cell proliferation and contributes to plant development. The proper re-organization of the cytoskeleton, organelles, and endomembrane networks are essential for cell division. Before plant cells divide, chromosomes are duplicated while cells often reach a size threshold minimum (Sablowski, 2016; Jones et al., 2017; D’Ario et al., 2021). The nucleus also migrates towards the future division plane and influences the positioning of subsequent mitotic cytoskeletal structures (Facette and Smith, 2012; Wada, 2017; Facette et al., 2019). In addition to interphase microtubules and the mitotic spindle, plants have two additional microtubule structures: the preprophase band (PPB) and the phragmoplast. The PPB comprises a ring of microtubules, and actin filaments that accumulate at the cell cortex before mitosis (Pickett-Heaps and Northcote, 1966). Many proteins additionally co-localize with the PPB, including microtubule-binding proteins (Van Damme et al., 2004). The PPB is not required for divisions, but when formed, it accurately predicts the future division site in many land plant divisions (Rasmussen and Bellinger, 2018; Livanos and Müller, 2019). The location of the PPB is just under the plasma membrane, known as the cortical division zone (Van Damme and Geelen, 2008; Smertenko et al., 2017). In telophase, the phragmoplast, a microtubule and microfilament structure, forms from spindle remnants (Lee and Liu, 2013; Smertenko, 2018). The phragmoplast directs formation of the cell plate during cytokinesis (Gu and Rasmussen, 2022; Sinclair et al., 2022). Cell plate assembly requires movement of vesicles along the phragmoplast, as well as vesicle fusion in the phragmoplast midline (Jürgens et al., 2015; Müller and Jürgens, 2016).

Cellular functions, such as chromosome separation during mitosis and formation of the new cell wall during cytokinesis, depend on proper regulation of microtubule dynamics. Microtubule dynamics are modulated by microtubule-associated proteins (MAPs) (Hashimoto, 2015). A subset of functionally diverse MAPs that bind to the plus end of microtubules are called plus-end-tracking proteins. The conserved eukaryotic protein END-BINDING PROTEIN (EB1) is a microtubule-plus-end tracking protein that binds growing microtubule ends, interacts with other proteins, and stabilizes the plus end (Van Damme et al., 2004; Nehlig et al., 2017). In *Arabidopsis thaliana*, EB1 localizes to microtubule plus ends and accumulate in mitotic structures (Chan et al., 2003; Mathur et al., 2003; Dixit et al., 2006; Bisgrove et al., 2008; Komaki et al., 2010).

A critical change that occurs before the onset of mitosis is the movement of the nucleus either to the middle of the cell for a symmetric division or towards one side of the cell during asymmetric division. Premitotic nuclear positioning in *A. thaliana* stomatal precursor cells depends on microtubules (Muroyama et al., 2020). In contrast, actin filaments, but not microtubules, are essential for premitotic nuclear migration of the first division of the *A. thaliana* zygote (Kimata et al., 2016). Actin-based nuclear movement also occurs prior to asymmetric divisions during stomatal development in maize (Kennard and Cleary, 1997). Nuclear migration is partially dependent on the interaction of actin with a protein in the linker of nucleoskeleton and cytoskeleton (LINC) complex in *A. thaliana* called SINE2 and in maize called MAIZE LINC KASH SINE-LIKE2 (MLKS2) (Gumber et al., 2019b), (Zhou et al., 2014). In maize, *mlks2* mutants have asymmetric division defects (McKenna et al., 2021) due to defective nuclear positioning that causes defects in PPB positioning (Arif Ashraf et al., 2022). A typical live cell marker used to explore nuclear dynamics and chromosome movement in *A. thaliana* is HISTONE2B fused to Yellow Fluorescent Protein (H2B:YFP) (Boisnard-Lorig et al., 2001). In maize, HISTONE2B fused to m-Cherry labels chromosomes in mitotic, interphase, and meiotic cells (Howe et al., 2012). The HISTONE1.1-YFP marker described below allows additional flexibility in combination with other fluorescent marker lines.

The nuclear envelope can be visualized by the localization of RAN GTPase activating Protein1 (RANGAP1). RANGAP increases RAN GTPase activity to generate the inactive GDP-bound form. In *A. thaliana*, RANGAP1 (AT3G63130) localizes to the division site throughout mitosis and cytokinesis (Xu et al., 2008). RANGAP1 performs essential GAP functions redundantly with RANGAP2 (Xu et al., 2008). The GAP activity of RANGAP1 is essential while its localization to mitotic structures and the division site is dispensable (Boruc et al., 2015). In contrast to continuous localization of RANGAP1 in *A. thaliana* at the division site, in onion cells, RANGAP1 is localized just below the PPB towards the cytoplasmic side. Further, onion RANGAP1 is not retained at the division site during the transition to metaphase (Yabuuchi et al., 2015).

During metaphase, the dynamic movement of organelles and subcellular structures promotes their proper segregation into daughter cells. The spindle assembles after nuclear envelope breakdown in metaphase (Dixit and Cyr, 2002) and chromosomes are separated in anaphase. On entry into metaphase, the endoplasmic reticulum (ER) dynamically reorganizes to the spindle poles, observed both using electron microscopy (Porter and Machado, 1960) and confocal microscopy of live cells with ER-retained HDEL-GFP (Nebenführ et al., 2000; Gupton et al., 2006). During division, ER organization is thought to be mediated by microtubules, whereas in interphase cells, plant ER organization primarily depends on interactions with actin microfilaments (Zachariadis et al., 2003; Gupton et al., 2006). Two highly conserved proteins that label the ER are PROTEIN DISULFIDE ISOMERASE1 (PDI1) and GLOSSY8. PDI1 plays a crucial role in mediating disulfide bond formation required for proper protein folding within the ER lumen (Li and Larkins, 1996). In contrast, GLOSSY8 is a beta-ketoacyl reductase enzyme required for cuticular wax synthesis found within ER membrane fractions (Xu et al., 1997; Xu et al., 2002). These proteins label the ER lumen and ER membrane, respectively.

During cytokinesis, plasma membrane localized proteins are sometimes localized at the cell plate, potentially to aid partitioning into the plasma membrane after cytokinesis is completed. Two examples that localize to the cell plate are the related auxin efflux transporters PINFORMED1 (PIN1) and PIN2 in Arabidopsis. PIN1 localizes to the cell plate where it interacts with DYNAMIN RELATED PROTEIN1A (DRP1A) (Mravec et al., 2011). Similarly, Arabidopsis PIN2 localizes to the cell plate during late telophase (Men et al., 2008). In maize, two related kinase-dead receptor-like kinases have varied accumulation at the cell plate: PANGLOSS1 (PAN1) localizes to the cell plate, while another unrelated receptor-like kinase, PAN2, does not accumulate in the cell plate (Sutimantanapi et al., 2014). Together, this suggests that cell plate accumulation is a common, but not default localization, for plasma membrane proteins. PLASMA MEMBRANE INTRINSIC PROTEIN2 (PIP2), a protein that mediates water transport, localizes to the plasma membrane in maize (Zelazny et al., 2007; Berny et al., 2016; Martinez et al., 2018), but little is known about PIP2 localization during cytokinesis.

Other proteins that localize to the cell plate are essential for cell plate formation. These include SNARE proteins that facilitate vesicle docking and fusion. Mutations in genes that disrupt cell plate formation lead to defects in cytokinesis that result in lethality or tiny club-shaped seedlings (Söllner et al., 2002; Gillmor et al., 2016). KNOLLE is a cytokinesis-specific syntaxin that localizes to the cell plate during telophase (Lukowitz et al., 1996; Lauber et al., 1997). KNOLLE accumulates in small motile particles starting in late G2 as it is trafficked through the ER, the Golgi, and the Trans-Golgi Network (TGN) towards the cell plate (Reichardt et al., 2007; Karnahl et al., 2017). Once it localizes to the cell plate, KNOLLE forms SNARE complexes with the syntaxin regulator KEULE to promote proper fusion of Golgi-derived vesicles containing cargo used to construct the cell plate (Waizenegger et al., 2000). KNOLLE is recycled into vacuoles at the end of cytokinesis (Reichardt et al., 2007).

Another class of proteins, small GTPases in the Rab (Rat sarcoma (Ras) in brain, Rab) superfamily, play critical roles in vesicle transport and tethering throughout the endomembrane system and often localize to the forming cell plate (Elliott et al., 2020). The RAB-GTPases used in this study are RAB1A, RAB2A and RAB11D. Maize *RAB1A* is most closely related to *AtRABD2a* (Zhang et al., 2007; Okekeogbu et al., 2019), which is involved in ER to Golgi transport (Rutherford and Moore, 2002). The maize *RAB2A* was named to reflect its similarity to the yeast homolog *RAB2.* The closest homolog of maize *RAB2A* in *A. thaliana*, *RABB1c/RABB1^b^*, encodes a protein that co-localizes with the Golgi, but does not label the cell plate (Rutherford and Moore, 2002; Chow et al., 2008). Maize RAB2A is enriched in the Golgi fraction (Okekeogbu et al., 2019), however its subcellular localization using microscopy has not been determined. The two RAB-GTPases most closely related to maize RAB11D are *AtRABA4d* and *AtRABA4a*, which have specific roles in polarized growth in pollen tubes and root hairs, respectively. RABa4d localizes towards the growing tip in motile particles. *raba4d* mutant pollen tubes grow abnormally, have reduced pectin accumulation, and result in reduced fertility *via* improper pollen tube guidance (Szumlanski and Nielsen, 2009; Zhou et al., 2020). In Arabidopsis and tobacco, the *RAB11D* homologs encode proteins that localize to the TGN where the proteins are organized and packaged in preparation for delivery through the cell (Dunkley et al., 2006; Toyooka et al., 2009). In maize, RAB11D is enriched in the Golgi fraction, but its localization during division is unknown (Okekeogbu et al., 2019).

Here we characterize the localization of proteins and organelles during maize cell division using confocal microscopy and disruption of cell plate formation using two chemicals with distinct activities. Since these images were taken using confocal microscopy, we acknowledge that spatial resolution is limited. The well-described live cell microtubule marker lines (YFP-TUBULIN and CFP-TUBULIN) were imaged together with proteins that label microtubule plus ends, chromosomes, the ER, the plasma membrane, and the cell plate. We show that the ER aligns with mitotic structures. RAB-GTPases are required for vesicle-target docking, and the cytokinesis-specific syntaxin KNOLLE is required for vesicle fusion. Several RAB-GTPases and KNOLLE localize to the cell plate and motile particles. We incubated maize leaves with Endosidin 7 (ES7), a chemical that inhibits cytokinesis-specific callose deposition in Arabidopsis (Park et al., 2014) and disrupts cytokinesis in algae (Davis et al., 2020). ES7 pulse treatments did not affect accumulation of cell plate localized proteins or disrupt cell plate morphology in maize. Longer 5-day ES7 incubation did not cause obvious cell plate defects in maize roots. Maize epidermal cells were also treated with chlorpropham (CIPC), a herbicide that disrupts microtubules. CIPC treatment generated multiple phragmoplasts and fragmented the cell plate but did not typically alter protein accumulation at the cell plate. Together, these data provide a framework for understanding dynamic movement of organelles and proteins during mitosis and cytokinesis.

## Materials and methods

Maize plants were grown in 2-quart pots in standard greenhouse conditions (temperature setpoint between 31 - 33 C) with supplementary lighting ~400 µE m^-2^ s^-1^ from 1000Watt high pressure sodium bulbs (Gravita Pro Plus 1000W). Plants were grown for three to five weeks from seeds. Maize transgenic plants were identified by painting the leaf with 0.4% glufosinate (Basta Finale) in 0.05% tween or by genotyping using specific primers, listed in **Supplementary Table 1**. Leaf tissue was ground using a TissueLyser (Qiagen) for DNA extractions and PCR was performed using MyTaq (Bioline) or KOD Hot Start polymerase (EMD Millipore) according to manufacturer’s conditions supplemented with 7% (vol/vol) DMSO.

Microscopy was performed using an Eclipse TE inverted stand (Nikon) with a W1 spinning disk (Yokagawa), EM-CCD camera (Hamamatsu 9100c), standard solid-state lasers (Obis from 40 mW to 100 mW), and an ASI Piezo stage controlled with micromanager software (www.micromanager.org) built by Solamere Technology. Standard emission filters were used (Chroma Technology). For YFP–TUBULIN, PDI-YFP, YFP-KNOLLE, RANGAP-YFP, PIN1A-YFP, RAB1A-YFP, and RAB11D-YFP, a 514 nm laser with emission filter 540/30 was used. For CFP–TUBULIN, RAB1A-CFP, PIP2a-CFP, and aniline blue-stained samples, a 445 nm laser with emission filter 480/40 was used. For EB1-mCherry and GLOSSY8-RFP, a 561 nm laser with emission filter 620/60 was used. A 100X oil immersion lens (1.48 NA) was used with immersion oil (Type FF, Cargille) for leaf epidermal tissue and a 40X oil immersion lens (1.15 NA) was used with immersion oil (Series AAA 1.330 Refractive Index Liquid, Cargille) for root imaging.

**Figure 1:**
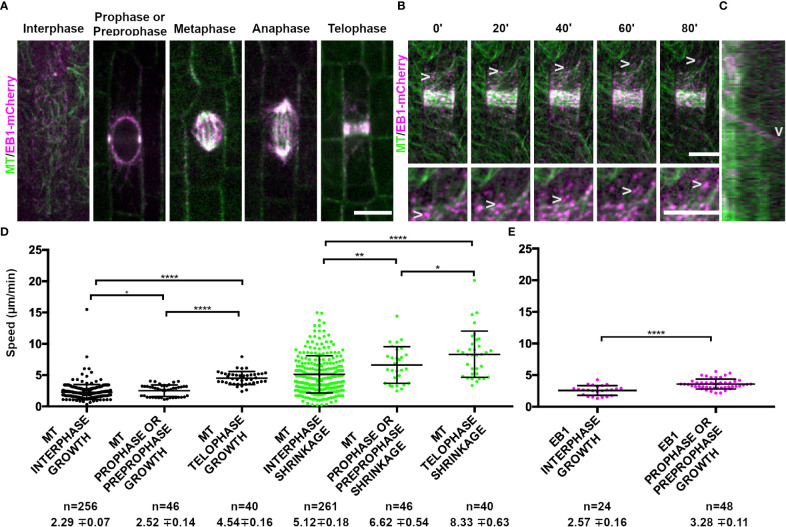
Microtubule binding protein END BINDING1 (EB1-mCherry) localizes to microtubule plus-ends and extensively co-localizes with YFP-TUBULIN during mitosis. Microtubules (false-colored green) from the abaxial side of maize leaves in regions with symmetrically dividing cells were imaged with YFP-TUBULIN and EB1 is false-colored magenta. **(A)** EB1-mCherry labels the plus-end of growing microtubules. **(B)** Timelapse of a cell in prophase. Arrowheads mark the EB1-mCherry protein. Zoom images of a prophase cell to visualize EB1. Arrowheads point at EB1-mCherry as the microtubule grows in the timelapse. Background subtraction was used in FIJI. Scale bars are 10µm. **(C)** Kymograph of EB1-mCherry protein tracking plus-end of microtubule. EB1-mCherry disappears followed by microtubule shrinkage (arrowhead). **(D)** Microtubule growth and catastrophe speed (µm/min) in wild-type maize leaf epidermal cells from 4 plants in interphase (n = 29 cells), prophase and preprophase (n = 2 cells) and telophase cells (n = 2 cells). **(E)** EB1 particle speed (µm/min) in wild-type maize leaf epidermal cells from 2 plants in interphase (n = 7 cells) and pre-prophase and prophase cells (n = 10 cells). P-values * < 0.05, ** < 0.01, **** < 0.0001 by Mann-Whitney *U* test.

Time-lapse and other imaging experiments were performed using a Rose chamber or glass slides, vacuum grease, and coverslips with a temperature between 20 - 22°C (Rasmussen, 2016). Three to five-week-old maize plants were used and leaves were removed until the ligule height was < 2 mm, and abaxial symmetrically dividing leaf epidermal samples were imaged. While imaging PIN1-YFP, the developing ligule was selected for imaging, as described (Neher et al., 2023). Samples were mounted in water. Mitotic structures were identified using a live cell marker for microtubules (either CFP-TUBULIN or YFP-TUBULIN) as previously described (Mohanty et al., 2009). Drift in the time lapse was corrected using the StackReg tool in FIJI (ImageJ) using the translation option (Thévenaz, 1998). Time intervals of 2.5 and 3 seconds were used for EB1-mCherry and YFP-TUBULIN microtubule time lapse imaging respectively. Kymographs were generated using Fiji’s Multi-kymograph tool to track EB1-mCherry particles and microtubules (Zanic, 2016). Mann-Whitney *U* test (GraphPad Prism) was used for statistical analysis for graphs in **Figure 1**.

Time intervals of 1 to 4 seconds were used to capture YFP-KNOLLE, RAB11D-YFP, RAB1A-CFP, and RAB2A-YFP particle trafficking. Initial imaging intervals of 4 seconds were used and then changed to 1 and 2 second intervals for more efficient particle tracking. Time intervals: YFP-KNOLLE: 2 and 4 seconds, RAB11D-YFP: 2 and 3 seconds, RAB1A-CFP: 2 seconds, RAB2A-YFP: 1 and 2 seconds. 30-118 seconds timelapses were used for particle tracking. Particles were tracked with the FIJI plugin Mosaic with the following parameters: kernel radius = 2.0, cutoff radius = 0.2, percentile = 0.5, displacement = 10, link range = 2 and Brownian Motion (Sbalzarini and Koumoutsakos, 2005). The particle coordinates were exported onto Excel or Google Sheets and converted from pixels to microns. The slope of the particle movement between frames was calculated using the slope formula (m = (y_2_-y_1_)/(x_2_-x_1_)) before taking the absolute value of all the numbers. The values were then divided by the interval of the time lapse (in seconds) to account for the time elapsed between each frame. Afterwards, the speeds were averaged to generate an average speed of that particle. The particle speeds between non-telophase and telophase stages within the same marker and across markers were compared using a t-test with the Bonferroni correction.

Fluorescence intensity of YFP-KNOLLE, RAB11D-YFP, RAB2A-YFP and RAB1A-CFP was measured using a set ROI in Fiji based on the size of the smallest particle, a RAB2A particle. To avoid the photobleached parts of the time lapse or changes in the intensity, measurements were only taken from one frame per image. Five sets of data points were measured in different parts of the cell, including the background, cell plate, and particles found in dividing and non-dividing cells, respectively, and then averaged. The average of the cell plate or particle was subtracted from the average of the background, respectively. A log of the ratio of the cell plate and particle fluorescence intensities was generated to determine whether the cell plate or the particles had stronger fluorescence intensity.

For maize pulse-treatment with CIPC and Endosidin 7 (ES7), matched samples were used in which one side of each leaf (from the midrib) was incubated with the treatment, while the other side was incubated with a corresponding amount of DMSO. For CIPC treatments, 4-week-old plants were used, and dissections were taken from symmetrically dividing leaf blades where the ligule was less than 2 mm in height. Leaf sections were then placed in a coverslip with either 20 µL of 0.02% DMSO or 2 µM CIPC for 2-4 hours at room temperature (~21 °C). For treatment with ES7, 1 mM ES7 was used, and matched negative control samples were incubated with DMSO (2%). Samples were then loaded into a Rose Chamber and the abaxial side was imaged.

To measure the long-term effect of ES7 on maize roots, maize kernels were germinated for 5 days between two layers of germination paper soaked in 50 ml of 0.02% DMSO (negative control) or 10 µM ES7 in 6-quart bins (Sterilite). Three technical replicates were done, where one secondary root was chosen from 4 plants for each treatment. Secondary roots were imaged because primary roots were too thick to effectively image. Roots were stained for 5 minutes in propidium iodide (10μg/mL, Fisher Scientific) and loaded into a Rose chamber for imaging using a 40X objective (NA = 1.15).

For *Arabidopsis thaliana* ES7 treatments, wild-type (Col-0/Ws) seeds were sterilized with chlorine gas for 2 h at room temperature (∼21°C) (Lindsey et al., 2017). ¼ strength MS media (Murashige and Skoog, 1962), 1% sucrose, pH 5.7, 0.8% (w/v) agar plates with either no treatment, 10 µM in 0.02% DMSO Endosidin7, or 0.02% DMSO, were used. Plants were placed in the dark for 2 days at 4 °C and then moved to the light for 5 days at 21 °C. After staining for 1 minute in propidium iodide (10 μg/mL), seedling roots were imaged using a Rose chamber and 40x objective in 20 µl H_2_O. To measure root lengths of ES7 and DMSO treated samples, 40 plants per treatment from two separate replicates were measured using the segmented line tool in FIJI. Datapoints were pooled after determining that there was no significant difference between replicates using Mann-Whitney *U* test. Pulse treatments were done using wild-type plants (Col-0/Ws) with CFP-TUBULIN grown on ¼ MS plates for 5 days with no treatment, then transferred into 2 ml of ¼ MS with either 50 µM ES7 or 0.1% DMSO treatment for 2 hours. After staining for 30 minutes in FM4-64 (2 µM), 3 replicates of 3-4 seedling roots were imaged. Three different cell plate morphologies were observed: normal, gap (gaps in the cell plate), and stub (when the cell plate is incomplete).

To assess the accumulation of callose in cell plates during pulse treatments, four day old Arabidopsis Col-0 seedlings were treated with 50 µM ES7 (ChemBridge Corporation, San Diego, CA, USA) in 0.1% DMSO in ¼ Murashige and Skoog (MS) liquid medium, or 0.1% DMSO in ¼ MS liquid medium for two hours (Park et al., 2014) and imaged as described below. For FM4-64 imaging, 4-d-old seedlings were incubated for 5 min in the dark with ¼ MS medium supplemented with 2 μM FM4-64, followed by a quick washing step in FM4-64–free medium (Rigal et al., 2015; Rosquete et al., 2019). Callose staining was performed in 0.03 mg/mL dilution of Aniline Blue fluorochrome in water for 3-5 minutes (Biosupplies, Melbourne, Australia), washed once in water, and directly imaged (Davis et al., 2020). A Zeiss LSM 980 Airyscan 2 was used in Airyscan Fast mode (SR8Y) to image root cells co-stained with Aniline Blue and FM4-64 following chemical treatment. For multicolor imaging, the sequential line-scanning mode was employed. Fluorescent signal of FM4-64 was excited using a 488 nm laser and emission was collected above 493 nm using a plate as SBS. Aniline blue fluorochrome was excited with a 405 nm laser and emission was collected with a SP 550 nm SBS. The Airyscan GaAsP-PMT detector was used with an offset set of 0, a detector gain of 850V and a digital gain of 1.0. All images were collected using the LD LCI Plan -Apochromat 40X/1.2 Imm Korr M27 objective. Z-stacks were collected using bidirectional scanning.

To assess the accumulation of callose in maize, callose staining was performed as described (Zavaliev and Epel, 2015). CIPC, ES7, or DMSO treated samples that were imaged for either YFP-KNOLLE or RAB11D-YFP were subsequently fixed in 96% ethanol for three hours. Samples were rehydrated in deionized water for 30 minutes. Samples were then stained with 0.01% aniline blue by vacuum infiltration (-30 kPa for 10 minutes), then incubated at room temperature in the dark for two hours. Tiled imaging was used to sample evenly and to prevent bias. Samples treated with either CIPC, ES7 or DMSO were imaged to 1) observe the presence of callose in cell plates and 2) observe the morphology of the cell plate present in the samples. Three different cell plate morphologies were observed: normal, stub (when the cell plate is incomplete), and split (a cell plate with multiple ends).

The EB1-mCherry construct was assembled using triple template PCR (KOD hot start, Sigma Aldrich) to generate the full genomic sequence (primers ZmEB1A_3GWp1.3:

GGGGACAAGTTTGTACAAAAAAGCAGGCTCAGAGCACAGGCAAGAGTGG and ZmEB1A_3GWp4 GGGGACAACTTTGTATAGAAAAGTTGGGTGCTCGGTTTCATTTGAGAACAAGC, and ZmEB1A_3GWp3 GGGGACAACTT GTATAATAAAGTTGAGTGAGATGTGCGGCTACATGA and ZmEB1A_3GWp2 GGGGACCACTTTGTACAAGAAAGCTGGGTAGAAAGCCGTATTGGCATCAC) with the m-Cherry insert (in pDONR P3r-P4r) at the C terminal end, flanked by linker peptides to minimize folding interference. The PCR products were cloned using the Gateway system (Gateway LR Clonase II Plus, Invitrogen) into the donor vectors, pDONR P1-P4 and pDONR P3-P2.

YFP-KNOLLE was generated by a 939-bp genomic DNA fragment including the entire KNOLLE coding region and 5.7 kb of 5′ sequence amplified from B73 genomic DNA with primers KNOLLE-3GWp1 (Primer sequence = GGGGACAAGTTTGTACAAAAAAGCAGGCTCAGAGAGGAGGTGACCAAGC) and Knolle-3GWp4 (Primer sequence = GGGGACAACTTTGTATAGAAAAGTTGATCCAAATCTACAACCGGCAGG). A 305-bp fragment immediately 3′ of the KNOLLE coding region was amplified from B73 genomic DNA with KNOLLE-3GWp3 (Primer sequence = GGGGACAACTT GTATAATAAAGTTGATGAACGACCTCATGACCAAGTCCTTCATGAGC) and KNOLLE -3GWp2 (Primer sequence = GGGGACCACTTTGTACAAGAAAGCTGGGTATCCAGTGATCGGCACTATG). Citrine variant YFP was amplified as described previously (Mohanty et al., 2009). These three fragments were assembled in pDONR221 (Invitrogen) to insert YFP in frame with KNOLLE at its N terminus with the 3′ KNOLLE flanking sequence downstream using a MultiSite Gateway three-fragment vector (Invitrogen) following the manufacturer’s instructions. Both EB1-mCherry and YFP-KNOLLE constructs were recombined into the binary vector pAM1006 (Mohanty et al., 2009), transformed into *Agrobacterium tumefaciens* strain EHA101 and transformed into maize A188/B73 hybrid embryo callus by the Iowa State Plant Transformation Facility. Transformed plants were crossed into the inbred line B73.

## Results

The conserved microtubule plus end localized protein, END-BINDING1 (EB1; Zm00001eb068860), fused to a monomeric red fluorescent protein (EB1-mCherry), localized to all mitotic structures (**Figure 1A**) and labeled microtubule plus ends (arrowheads, **Figure 1B**). For additional information about genes, including likely orthologs in Arabidopsis, predicted or known localization, and additional references, see **Table 1**. As expected, EB1-mCherry localization tracked the microtubule plus end, but was lost when the microtubule started shrinking (**Figure 1C**). Interestingly, both microtubule growth and shrinkage speeds increased from interphase (growth = 2.29 ± 0.07 µm/min standard deviation (SD), n = 256 microtubules; shrinkage = 5.12 ± 0.18 µm/min SD, n = 261 microtubules, 4 plants each) to prophase (growth = 2.52 ± 0.14 µm/min SD, n = 46 microtubules; shrinkage = 6.62 ± 0.54 µm/min SD, n = 46 microtubules, 2 plants each) to telophase (growth = 4.54 ± 0.16 µm/min SD, n = 40 microtubules; shrinkage = 8.33 ± 0.63 µm/min SD, n = 40 microtubules, 2 plants, **Figure 1D**). Similar increases in EB1-mCherry particle movement was also detected between interphase (2.57 ± 0.16 µm/min SD, n = 24 microtubules, 3 plants) and prophase (3.58 ± 0.11 µm/min SD, n = 48 microtubules, 3 plants, **Figure 1E**). We were unable to assess EB1-mCherry dynamics in metaphase or anaphase because the EB1-mCherry particle density was too high in the spindle to clearly track. Together, this suggests that EB1 dynamics are similar to microtubule dynamics, and that microtubule dynamicity increases as the cell progresses through mitosis, similar to observations in tobacco cultured cells (Vos et al., 2004).

**Table 1:** The gene name and ID used in this study and the putative Arabidopsis ortholog, and references.

Gene name and ID	Putative Arabidopsis orthologous gene id	Predicted or known localization and function	Reference
*END BINDING1*, Zm00001eb068860 GRMZM5G824964	*EB1a* AT3G47690	Microtubule plus ends. Stabilizes microtubules and to mediates interactions with other microtubule binding proteins.	(Chan et al., 2003; Mathur et al., 2003; Van Damme et al., 2004; Dixit et al., 2006; Bisgrove et al., 2008; Komaki et al., 2010)
*HISTONE1.1*, Zm00001d034479 GRMZM2G164020	*HISTONE1.2* AT2G30620	Chromosomes. Linker histone H1a, interacts with ZmSUN2 by IP/MS.	(Boisnard-Lorig et al., 2001; (Gumber et al., 2019a)
*RANGAP1*, Zm00001d051112 GRMZM2G079817	*RANGAP1* AT3G63130	Nuclear envelope, division site. GTPase activating protein for RAN monomeric GTPase.	(Xu et al., 2008; Yabuuchi et al., 2015)
*GLOSSY8*, Zm00001eb246270	*KCR1* AT1G67730	Endoplasmic reticulum membranes. Very long chain fatty acid production, beta-ketoacyl reductase.	(Xu et al., 1997; Xu et al., 2002; Dunkley et al., 2006; Kirienko et al., 2012; Okekeogbu et al., 2019)
*PDI1*, Zm00001eb168910, GRMZM2G091481	*PDI1* AT1G21750	Endoplasmic reticulum lumen. Isomerase.	(Li and Larkins, 1996; Dunkley et al., 2006; Kirienko et al., 2012)
*KNOLLE*, Zm00001d033919	*KNOLLE* AT1G08560	Cell plate. Syntaxin, SYP111.	(Lukowitz et al., 1996)
*RAB11D*, Zm00001d028002 GRMZM2G164527	*RABA4a* AT5G65270	TGN, post-Golgi vesicles, plasma membrane. Monomeric GTPase involved in vesicle trafficking.	(Vernoud et al., 2003; Dunkley et al., 2006; Szumlanski and Nielsen, 2009; Okekeogbu et al., 2019)
*RAB1A*, Zm00001d017456; GRMZM2G097746	*RABD2a* AT1G02130	Membranous particles distinct from FM4-64 in Arabidopsis. Required for pollen tube growth.	(Peng et al., 2011; Okekeogbu et al., 2019)
*RAB2A*, Zm00001eb080090 GRMZM2G330430	*RABB1C or RABB1^b^* AT4G17170	Golgi localization, does not localize to the cell plate in Arabidopsis.	(Chow et al., 2008; Okekeogbu et al., 2019)
*PIP2-1*, Zm00001eb306380	*PIP2;4* AT5G60660	Localizes to the plasma membrane in maize. Aquaporin, water transport.	(Zelazny et al., 2007; Berny et al., 2016; Martinez et al., 2018)
*PIN1*, Zm00001eb372180	*PIN1* AT1G73590	Auxin efflux transporter, localizes to the plasma membrane.	(Mravec et al., 2011)

**Figure 2:**
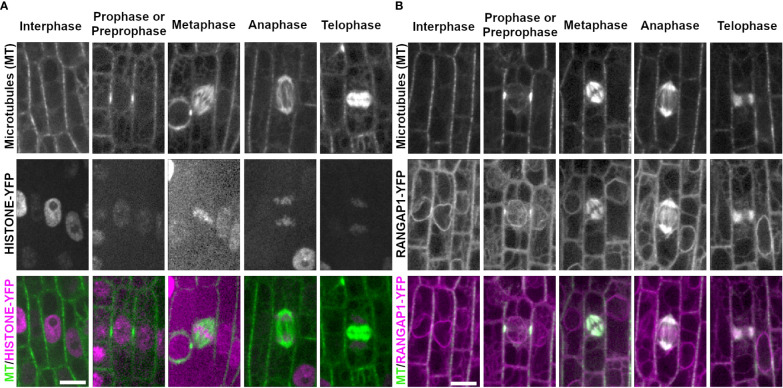
Chromosome marker HISTONE1.1-YFP and nuclear envelope marker RANGAP1-YFP with CFP-TUBULIN during the cell cycle. Microtubules from the abaxial side of maize leaves in regions with symmetrically dividing cells were imaged with CFP-TUBULIN (top row). In the merged images, microtubules are colored green, while HISTONE1.1-YFP or RANGAP1-YFP are colored magenta. **(A)** Chromosomes are labeled with HISTONE1.1-YFP (middle row). **(B)** RANGAP1-YFP labels the cell periphery, the nuclear envelope during interphase and preprophase/prophase, and localizes close to the PPB during prophase/preprophase. During mitosis, RANGAP1-YFP evenly labels all microtubule structures but is not at the division site. Preprophase/prophase RANGAP1-YFP and CFP-TUBULIN images are maximum-projections of Z stacks covering 7.5 microns to more clearly illustrate RANGAP1-YFP localization near the PPB. Scale bar is 10µm, all images are the same size.

We next examined chromosome movement during mitosis using the chromosome binding protein HISTONE1.1 fused to a yellow fluorescent protein (HISTONE1.1-YFP, Zm00001e006785**)**. HISTONE1.1-YFP localized to the nucleus during interphase (n ≥ 100 cells, 2 plants) and prophase (n = 24 cells), specifically labeling chromosomes (**Figure 2A**). As expected, chromosomes accumulated at the metaphase plate (n = 10 cells), were separated during anaphase (n = 9 cells) and were in the nucleus when the nuclear envelope re-formed during telophase (n = 14 cells). The HISTONE1.1-YFP signal intensity varied extensively and was sometimes too faint to image clearly in mitotic cells. However, the location of chromosomes and nuclei can be inferred from other marker lines described below.

The nuclear envelope protein RANGAP1 fused to YFP (RANGAP1-YFP, Zm00001d051112) labeled the nuclear envelope and the cell periphery during most cell cycle stages in maize epidermal leaves (**Figure 2B**), as previously described (Wu et al., 2013; Arif Ashraf et al., 2022). During preprophase or prophase, RANGAP1-YFP uniquely labeled the region directly adjacent to the PPB away from the plasma membrane. RANGAP1-YFP labeled all mitotic structures as well (**Figure 2B**). During late G2 (preprophase) and prophase, RANGAP1-YFP localized close to the PPB, slightly offset towards the cytoplasmic side (91%, n = 43/47 cells from 2 plants, **Supplementary Figure 1**). Further, RANGAP1-YFP always labeled the nuclear envelope during interphase, preprophase, and prophase. After nuclear envelope breakdown, RANGAP1-YFP no longer labeled the cortical division zone, but consistently co-localized with the entire metaphase spindle (n = 13 cells), the anaphase spindle (n = 4 cells) and the phragmoplast (n = 23 cells). This localization pattern is similar to that observed with immunolocalization of RANGAP1 in onion root cells (Yabuuchi et al., 2015). Similar localization patterns within both maize and onion RANGAP1 suggests potentially conserved monocot function that may diverge from dicot RANGAP1, which localizes consistently at the division site in Arabidopsis (Xu et al., 2008).

**Figure 3:**
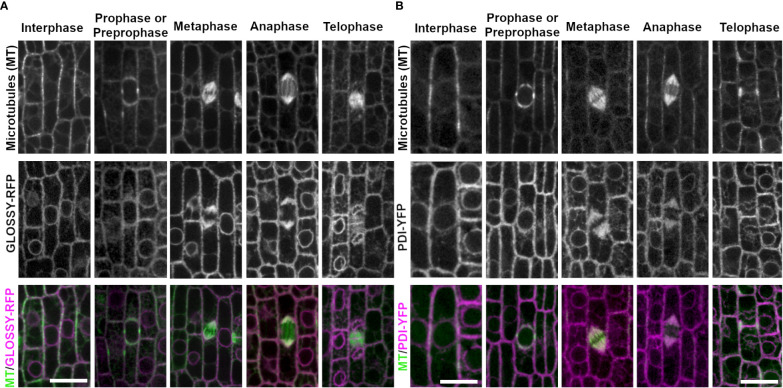
ER membrane marker GLOSSY8-mRFP and ER lumen marker PDI1- YFP with CFP-TUBULIN. Microtubules from the abaxial side of maize leaves in regions with symmetrically dividing cells were imaged with CFP-TUBULIN (top row). Microtubules are labeled green, while GLOSSY8-mRFP and PDI1-YFP are labeled magenta, in the merged photos (bottom row). **(A)** Endoplasmic reticulum membrane is labeled with GLOSSY8-mRFP. GLOSSY8-mRFP co-localizes with distal spindle regions during metaphase and anaphase. GLOSSY8-mRFP accumulates at the cell plate during telophase. **(B)** PDI1-YFP localization labels the endoplasmic reticulum lumen. PDI1-YFP localizes to distal spindle regions during metaphase and anaphase. Accumulation of PDI1-YFP is present near or in the cell plate and in the phragmoplast during telophase. Scale bar is 10µm.

Several proteins that label the endoplasmic reticulum (ER) accumulate near mitotic structures. To assess ER localization during mitosis, PROTEIN DISULFIDE-ISOMERASE 1 fused to YFP (PDI1-YFP; (Kirienko et al., 2012)), an enzyme within the ER lumen (Li and Larkins, 1996), and GLOSSY8-mRFP (Kirienko et al., 2012), an enzyme isolated from ER membranes (Xu et al., 2002), were imaged together with CFP-TUBULIN (**Figure 3**). In epidermal cells within the proliferative dividing zone, interphase PDI and GLOSSY8 localization appeared around the cell periphery and the nucleus, highlighting typical ER localization in this type of cell (**Figure 3A, B**, n = > 100 cells, 5 plants each). In premitotic and mitotic cells, PDI accumulated at the nuclear envelope when it was intact (n ≥ 100 cells). PDI1-YFP labeled a region just distal to the spindle in metaphase (n = 19 cells) and anaphase (n = 13 cells). During telophase, it accumulated near the cell plate and co-localized with the phragmoplast (n = 34 cells, **Figure 3B**). GLOSSY8-mRFP localized similarly to PDI1-YFP during prophase (n = 52 cells), at distal spindle regions in metaphase (n = 17 cells) and anaphase (n = 14 cells) and accumulated near the cell plate in telophase (n = 30 cells) (**Figure 3A**).

**Figure 4:**
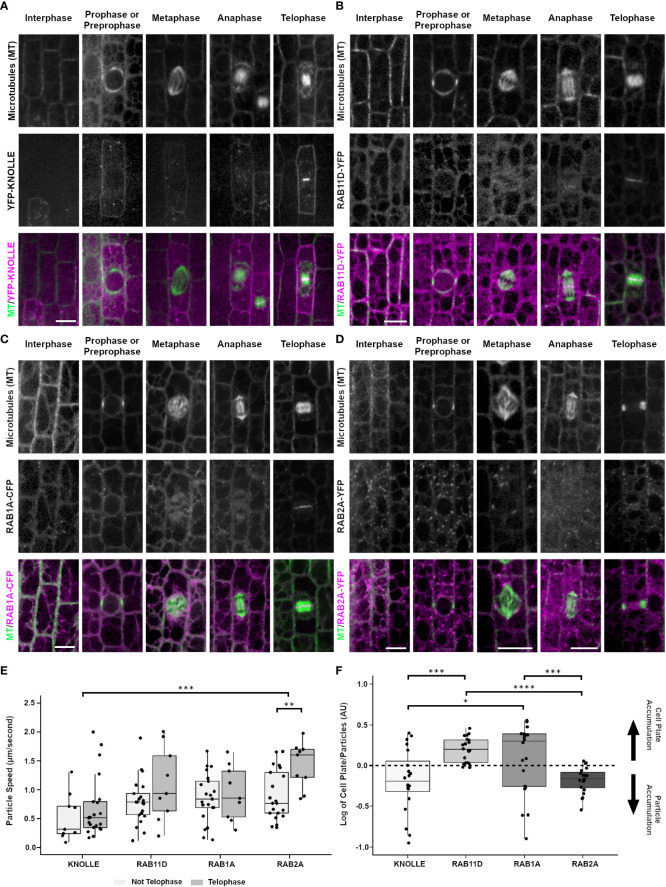
Localization of cell plate specific syntaxin KNOLLE (YFP-KNOLLE), likely trans-golgi marker RAB11D (RAB11D-YFP), vesicle tethering protein RAB1A (RAB1A-CFP), and golgi marker RAB2A (RAB2A-YFP) from the abaxial side of maize leaves in regions with symmetrically dividing cells. Microtubules (top row), marker (middle) and merged (bottom, microtubules in green and marker in magenta). Microtubules were imaged with CFP-TUBULIN in **(A, B, D)**. Microtubules were imaged with YFP-TUBULIN in **(C)**. **(A)** YFP-KNOLLE accumulates in motile particles, the plasma membrane and cell plate in mitotic cells. **(B)** RAB11D-YFP localizes as motile particles at all stages and accumulates in the cell plate. **(C)** RAB1A-CFP accumulates in motile particles and in the cell plate at telophase. **(D)** RAB2A-YFP accumulates in motile particles throughout interphase and mitosis. During telophase, RAB2A-YFP weakly accumulates in the cell plate. Scale bars for panels **(A-D)** are 10µm; if unlabeled, the micrograph has the same scale as the interphase cell. **(E)** Particle speeds of YFP-KNOLLE, RAB11D-YFP, RAB1A-CFP, and RAB2A-YFP in telophase vs. non-telophase cells. A t-test with Bonferroni Correction of the various marker comparisons shows that there are no significant differences in particle speeds in interphase cells. For dividing cells, there are no significant differences in particle speeds besides KNOLLE and RAB2A, ***p < 0.001, and RAB2A telophase and non-telophase cells **p < 0.01. **(F)** Relative fluorescence accumulation of YFP-KNOLLE, RAB11D-YFP, RAB1A-CFP and RAB2A was measured in cell plates versus in particles. 20 cell plates and 100 particles were measured for each marker from at least four plants. After determining that datasets were normally distributed (Jarque-Bera test) one-way Anova tests with the Bonferroni Correction were used to identify significant differences in relative fluorescence accumulation at the cell plate or particles between KNOLLE and RAB11D, RAB11D and RAB2A, as well as RAB1A and RAB2A. *p < 0.05, ***p < 0.001, ****p < 0.0001. Other comparisons had no significant differences in their fluorescence intensity log ratios.

Next, we examined several proteins that accumulate in the cell plate, including the cell plate specific syntaxin KNOLLE fused to YFP (YFP-KNOLLE, Zm00001d033919, **Figure 4A**). YFP-KNOLLE accumulated only during mitosis and early G1, with undetectable fluorescence accumulation in interphase cells (n ≥ 100 cells, n = 3 plants). YFP-KNOLLE localized throughout the cell as motile particles presumably labeling TGN during prophase (n ≥ 100 cells), metaphase (n = 31 cells) and anaphase (n = 30 cells, **Figure 4A**). The average speed of particles from prophase to anaphase was 0.53 µm/s ± 0.41 SD (n = 9 particles from 4 plants, 8 cells, **Figure 4E**). During telophase, YFP-KNOLLE accumulated strongly at the cell plate (5 plants, n > 100 cells). Distinct YFP-KNOLLE particles were also observed in telophase and had an average speed of 0.69 µm/s ± 0.53 SD, (n = 21 particles from 4 plants, 12 cells, and an example of particle movement is shown in **Supplementary Figure 2**). Occasionally, some of these particles moved into the cell plate. YFP-KNOLLE also faintly labeled the plasma membrane in mitotic cells. This is consistent with movement and localization of KNOLLE in Arabidopsis (Reichardt et al., 2007; Boutté et al., 2010).

Two monomeric GTPases, RAB11D fused to YFP (RAB11D-YFP, Zm00001d028002) and RAB1A fused to CFP or YFP (RAB1A-CFP or RAB1A-YFP, Zm00001d017456), localized as motile particles and in the cytoplasm in both interphase (5 and 3 plants respectively, n ≥ 100 cells) and mitotic cells (prophase n = 95 cells, metaphase n = 25 cells, anaphase n = 10 cells and prophase n = 25 cells, metaphase n = 12 cells, anaphase n = 13 cells respectively), and accumulated at the cell plate during telophase (n = 143 cells and n = 19 cells respectively, **Figures 4B, C**). The apparent diameter of RAB11D-YFP fluorescent foci were 0.81 µm ± 0.13 SD (n = 30 particles, 3 plants). RAB11D-YFP non-telophase motile particles moved with an average speed of 0.8 µm/sec ± 0.4 µm/s SD (n = 21 particles from 19 non-telophase cells from 5 plants, **Figure 4E**). During telophase, the average particle speed of RAB11D-YFP was 1.10 µm/sec ± 0.64 µm/sec SD, (n = 9 particles from 6 telophase cells from 5 plants) and there was strong cell plate accumulation. RAB1A-CFP also accumulated at the cell plate although fluorescence intensity measurements suggest that it accumulates more in motile particles (**Figure 4F**). Motile particles of RAB1A-CFP were found scattered throughout the cells between prophase and anaphase, but RAB1A-CFP also localized to the cell plate during telophase (**Figure 4C**). The apparent particle diameter was measured to 0.75 µm ± 0.11 SD (30 particles analyzed from 3 plants). RAB1A-CFP particle speed averaged 0.86 µm/sec ± 0.42 SD (n = 21 particles, 21 non-telophase cells from 3 plants, **Figure 4E**). RAB1A-CFP particles that localized to the cell plate during telophase had an average speed of 0.94 µm/sec ± 0.47 SD (n = 9 particles, 6 telophase cells from 3 plants, **Figure 4E**). Only RAB2A showed significant differences in particle speeds between telophase and non-telophase cells. Comparisons within and between the markers were done using an unpaired t-test with a Bonferroni correction. Fluorescence intensity measurements of YFP-KNOLLE and RAB2A-YFP show more accumulation at the particles than at the cell plate whereas RAB11D-YFP and RAB1A-CFP show more accumulation at the cell plate (**Figure 4F**). A total of 20 telophase cells were measured from 5 plants with YFP-KNOLLE, 5 plants with RAB11D-YFP, 5 plants with RAB2A-YFP, 7 plants with RAB1A-CFP.

Similar to the two monomeric GTPases discussed above, the monomeric GTPase RAB2A-YFP also localized to motile particles during interphase and mitosis (4 plants, interphase n ≥ 100 cells, prophase n = 87 cells, metaphase n = 24 cells, anaphase n = 26 cells, **Figure 4D**). In contrast to the other RAB-GTPases, RAB2A-YFP only faintly accumulated in the cell plate during telophase (n = 59 cells, 4 plants), suggesting that RAB2A-YFP may not be directly targeted to the cell plate (**Figure 4E**). In non-dividing cells, RAB2A-YFP particles had an average speed of 0.91 µm/s ± 0.43 SD (n = 21 particles from 21 cells from 3 plants). During telophase, RAB2A-YFP particles had an average speed of 1.43 µm/s ± 0.40 SD (n = 9 particles, 6 cells from 3 plants).

**Figure 5:**
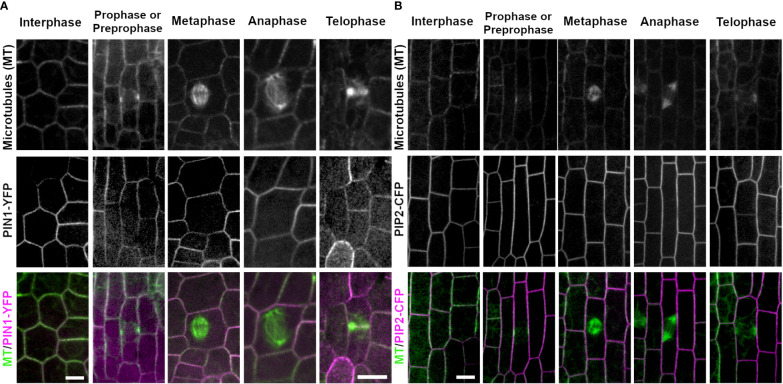
Plasma membrane markers PIN1 (PIN1-YFP) and PIP2 (PIP2-CFP). Microtubules were imaged with CFP-TUBULIN (left panels) and YFP-TUBULIN (right panels) in the top row. PIN1-YFP was imaged in the early fringe within the ligule and PIP2-CFP was imaged from the abaxial side of maize leaves in regions with symmetrically dividing cells in the middle row. Microtubules are labeled green and markers in magenta in the merged photos (bottom row). **(A)** PIN1-YFP localizes to the plasma membrane during interphase and all stages of mitosis. PIN1-YFP accumulates at the cell plate during telophase. **(B)** PIP2-CFP accumulates at the plasma membrane during interphase and all stages of mitosis in dividing epidermal tissue, and accumulates in the cell plate. Scale bars are 10µm.

PINIFORMED1 (PIN1) and PLASMA MEMBRANE INTRINSIC PROTEIN 2-1 (PIP2A) are both plasma membrane localized proteins that are required for transport of molecules across the plasma membrane. In all cell cycle stages examined, PIN1-YFP (3 plants, interphase, n ≥ 100 cells, prophase n = 19 cells, metaphase n = 14 cells, anaphase n = 5 cells, telophase n = 19 cells) and PIP2A-CFP (3 plants, interphase n ≥ 100 cells, prophase n = 33 cells, metaphase n = 22 cells, anaphase n = 6 cells, and telophase n = 39 cells) localized to the plasma membrane. In addition, during telophase, both PIN1-YFP and PIP2A-CFP weakly accumulated in the cell plate as compared to parental cell wall sites. Accumulation at each cell was measured as a fluorescence intensity ratio of fluorescence at the cell plate: fluorescence at the plasma membrane. The ratio was 0.794 ± 0.634 for PIP2-CFP (n = 3 plants, 13 cell plates) and 0.461 ± 0.375 for PIN1-YFP (n = 2 plants, 10 cell plates). PIN1-YFP accumulated in the cell plate 100% in both early telophase (n = 7/7 cells) and 90% in late telophase (n = 9/10 cells from 2 plants, **Figure 5A**). PIP2A-CFP also accumulated in cell plates, 91% in early telophase (n = 11/12 cells) and 100% in late telophase (n = 27 cells from 3 plants, **Figure 5B**).

**Figure 6:**
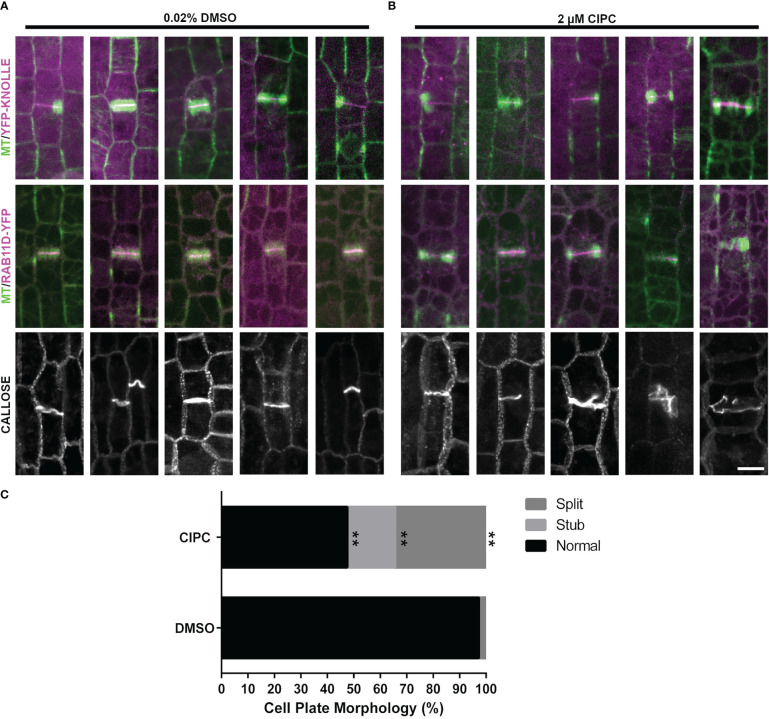
Effect of CIPC on the accumulation of YFP-KNOLLE, RAB11D-YFP, and callose to the cell plate. Microtubules imaged with the CFP-TUBULIN are labeled in green, with YFP-KNOLLE or RAB11D-YFP labeled in magenta, in the top and middle panels from the abaxial side of maize leaves in regions with symmetrically dividing cells. **(A)** Effect of 0.02% DMSO treatment on the accumulation of YFP-KNOLLE, RAB11D-YFP, and callose to the cell plate. **(B)** Effect of 2µM CIPC treatment on the accumulation of YFP-KNOLLE, RAB11D-YFP, and callose to the cell plate. **(C)** Percentage of cell plate morphology seen after callose staining on samples treated with either 0.02% DMSO or 2µM CIPC. DMSO: Stub = 0%, Split = 2%, Normal= 95%; CIPC: Stub = 18%, Split = 34%, Normal = 48%. Number of plants = 2, CIPC number of cells = 61, DMSO number of cells = 59. Scale bar is 10µm. For callose staining both YFP-KNOLLE and RAB11D-YFP number of plants = 3. In the YFP-KNOLLE plants cells in telophase n = 89 in the CIPC treatment, while for the DMSO treatment cells in telophase n = 37 cells. In the RAB11D-YFP plants, a total of telophase cells n = 36 were seen when treated with CIPC, while telophase n = 29 cells in the DMSO treatment. **p ≤ 0.01 unpaired t-test.

To determine whether cell-plate localization of the proteins described above was contingent on proper cell plate formation, maize epidermal cells were treated with the herbicide chlorpropham (CIPC, 2µM). CIPC binds tubulin and generates fragmented phragmoplasts and fragmented or split cell plates *in vivo* (Young and Lewandowski, 2000; Buschmann et al., 2006). CIPC indeed generated fragmented phragmoplasts and split cell plates. However, despite generating aberrant cell plates, two cell plate associated proteins, YFP-KNOLLE and RAB11D-YFP still accumulated normally at the cell plate (**Figures 6A, B**, YFP-KNOLLE (n = 3 plants, n = 88 cells with CIPC treatment, n = 38 cells with DMSO treatment) RAB11D-YFP (n = 3 plants, n = 29 cells with CIPC treatment, n = 36 cells with DMSO treatment). To further assess how CIPC disrupted cell plate formation, callose accumulation was visualized using aniline blue. Callose is a major polysaccharide deposited at the cell plate (Samuels et al., 1995). In CIPC treated samples, cell plate morphologies were often disrupted with approximately 50% of cells in telophase showing abnormal cell plates (n = 2 plants, n = 61 cells **Figure 6**). Abnormal cell plates observed in CIPC treatments were broken into three categories: cell plate stubs (n = 11/61 cells), split cell plates (n = 21/61 cells), and normal cell plates (n = 29/61 cells). In DMSO treated samples, cell plates were rarely disrupted (2% split cell plate, n = 1/59 cells, unpaired t-test, p-value < 0.01). These results indicate that CIPC does not disrupt the accumulation of vesicles and their cargo to the cell plate, but generated split and aberrant cell plates due to fragmented phragmoplasts.

Since CIPC disrupted cell plate morphology but did not alter accumulation of cell-plate associated proteins, we treated maize epidermal cells with Endosidin 7 (ES7) a drug that inhibits callose deposition at the cell plate, affects KNOLLE localization at late cell plate stages in Arabidopsis and disrupts cytokinesis in algae (Park et al., 2014; Davis et al., 2020). Maize epidermal cells were treated with different ES7 concentrations ranging from 25 µM to 1 mM. After 2-3 hours of incubation with ES7, we assessed YFP-KNOLLE accumulation at the cell plate (**Supplementary Figure 3A**). Fluorescence intensity measurements of YFP-KNOLLE in cell plates were similar between the negative control 2% DMSO treated plants (n = 3 plants, n = 21 cells) and 1 mM ES7 treated plants (n = 3 plants, n = 12 cells) and were not significantly different (Welch’s two sample t-test, p-value = 0.31, **Supplementary Figure 3C**). We further stained for callose in the ES7 treated plants (**Supplementary Figure 3A**, bottom panel). Callose staining patterns were not significantly different between cells treated with 1 mM ES7 for 3 hours (11.4% abnormal, n = 20/175 cells) and negative control treated with 2% DMSO (6.4% abnormal, n = 9/141 cells, Fisher’s exact test, p-value = 0.1694 (**Supplementary Figure 3D**).

In addition to pulse-treating maize leaf samples with ES7, we also grew maize seedlings for 5 days on germination paper supplemented with either 10 µM ES7 or 0.02% DMSO (**Supplementary Figure 4**). Root lengths of 10 µM ES7 treated plants (n = 3 replicates, 71 plants) were 5.6 cm ±1.6 and 5.8 cm ± 1.7 in the 0.02% DMSO negative control (n = 4 replicates, 58 plants, **Supplementary Figure 4A**). There was no statistically significant difference in the root length 5 days after germination between the two treatments (**Supplementary Figure 4B**, Mann-Whitney *U* test, p-value > 0.1). No cell wall stubs were seen in ES7 or DMSO treated maize roots stained with propidium iodide (**Supplementary Figure 2C**).

To ensure that we were using active and correctly diluted ES7, we used ES7 to treat Arabidopsis seedlings, as previously described (Park et al., 2014). The ES7 treatment slowed root growth and generated cell wall stubs, similar to previous reports (**Supplementary Figure 5**) (Park et al., 2014). We grew Arabidopsis (Col-0/Ws) seedlings for 5 days after stratification in 10 µM ES7. 10 µM ES7 treated plants had shorter roots (**Supplementary Figure 5A**) with an average of 0.18 cm ± 0.08 compared to the 0.02% DMSO negative control average length 0.81 cm ± 0.13 (**Supplementary Figure 5B**, Mann-Whitney *U* test, p-value < 0.0001). Arabidopsis seedlings were stained with propidium iodide and cell wall stubs were frequently observed in roots of seedlings grown in 10 µM ES7 for 5 days (**Supplementary Figure 5C**, right panel) in comparison to the 0.02% DMSO negative control (5C left panel). Alternatively, wild-type (Col-0/Ws) with CFP-TUBULIN seedlings were pulse-treated for 2 hours with 50 µM ES7 or 0.1% DMSO and stained with FM4-64 (2 µM) (**Supplementary Figure 5D**). When treated with 50 µM ES7, 19 incomplete cell plates or cell wall stubs were observed in 8 plants, versus 0 incomplete cell plates or cell wall stubs in 8 plants in 0.1% DMSO control (Fisher’s exact test, p < 0.05, 3 replicates, 8 plants, n = 0/1206 cells in 0.1% DMSO and n = 19/1055 cells in 50 µM ES7). Pulse treatment with 50 µM ES7 affected callose accumulation when compared to 0.1% DMSO in 4-day old seedlings (n = 10 seedlings per treatment, **Supplementary Figure 6**). There was no detectable callose deposition in aberrant cell plates (**Supplementary Figures 6D-F**). Overall, this indicates that ES7 affects Arabidopsis as expected, but does not affect maize roots or leaves with the conditions used here.

## Discussion

The localization of organelles, plasma membrane localized proteins, plus-end microtubule associated proteins, and proteins involved in vesicle transport are described in symmetrically dividing maize epidermal cells. First, we assessed how EB1, a protein that localizes to the plus-ends of microtubules, localizes in maize. Maize EB1 labels the growing plus-end of microtubules, disappearing as microtubules shrink. EB1 localization to microtubule plus-ends is similar to that seen in Arabidopsis, yeast, and human cells (Tirnauer and Bierer, 2000; Chan et al., 2003; Bisgrove et al., 2008; Komaki et al., 2010). Maize contains two *EB1* homologs (Zm00001eb068860 and Zm00001eb044540) which encode proteins with 64% amino acid identity. Both maize *EB1* homologs are most similar to *AtEB1a* and *AtEB1b.* Maize EB1(Zm00001eb068860) localization is more similar to AtEB1a and AtEB1b than AtEB1c, which localizes conspicuously in the nucleus in addition to the microtubule plus end (Komaki et al., 2010). Recently, maize EB1 (Zm00001eb068860) was shown to interact with TUBULIN FOLDING COFACTOR B, a protein that promotes tubulin folding and dimerization. Maize EB1 localized to plus-ends when expressed in *A. thaliana* protoplasts (Zhou et al., 2023). EB1 binds to and stabilizes an extended region past the microtubule plus end tip consisting of GTP-GDP-Pi-microtubules (Nehlig et al., 2017). Both EB1 and microtubule dynamics increased in cells in late G2 and prophase containing a PPB. Microtubule dynamicity continued to increase in telophase cells. In Arabidopsis, interphase microtubule plus-end growth rates in epidermal cells are ~3.7 µm/min (Shaw et al., 2003) and similar in cultured Arabidopsis cells ~3.5 µm/min (Chan et al., 2003). Maize interphase microtubule growth rates are slower, ~2.6 µm/min but both growth and shrinkage rates increased as cells formed PPBs and entered mitosis. Similar increased dynamicity is observed in cultured tobacco (BY-2) cells when cells had PPBs (Vos et al., 2004). In addition, faster growth and shrinkage was seen during telophase in maize epidermal cells, similar to previous reports (Bellinger et al., 2023). This increased microtubule dynamicity during mitosis may reflect alterations in the balance of microtubule associated proteins or in the relative amount of tubulin captured in various mitotic structures.

Maize ER and nuclear-envelope localized proteins dynamically repositioned during mitosis, similar to previous reports using live cell markers in Arabidopsis and tobacco cultured cells (Gupton et al., 2006; Oda and Fukuda, 2011; Meier et al., 2017). ER accumulation near mitotic structures has also been observed in monocots using transmission electron microscopy (Porter and Machado, 1960; Pickett-Heaps and Northcote, 1966). While maize ER and nuclear envelope localized proteins have similar localization during interphase, localization differences between RANGAP1, PDI1 and GLOSSY8 occur mainly during late G2 and mitosis. While RANGAP1 evenly labels the spindle, both PDI1 and GLOSSY8 accumulate strongly at spindle poles, but do not strongly accumulate at the spindle midzone. In Arabidopsis, RANGAP labels the division site, and kinetochores, but does not label the spindle poles (Xu et al., 2008). However, its localization is dispensable for function, as tested by removing motifs required for interaction with nuclear envelope proteins (Boruc et al., 2015). In both onion epidermal cells and maize, RANGAP1 localizes to a region just inside the PPB, but disappears from the division site on entry into metaphase (Yabuuchi et al., 2015). The function of the two ER-localized proteins examined here, PDI1 and GLOSSY8, have been well characterized in maize and Arabidopsis (Li and Larkins, 1996; Xu et al., 1997; Lu and Christopher, 2008; Kirienko et al., 2012).

Many of the proteins examined here localize to the cell plate during telophase and cytokinesis and to motile particles that may be Golgi, *Trans*-Golgi Network (TGN), or vesicle populations. We used the well-characterized YFP-KNOLLE to assess cell plate formation and associated vesicle trafficking. In Arabidopsis, this mitotic syntaxin mediates homotypic cell fusion: *knolle* mutants have cytokinesis defects and an accumulation of unfused vesicles at the cell plate (Lukowitz et al., 1996; Lauber et al., 1997). KNOLLE is expressed solely during mitosis (Lauber et al., 1997). Similarly, maize YFP-KNOLLE was not observed in interphase cells but accumulated as motile particles during mitosis that then accumulated in the developing cell plate. YFP-KNOLLE accumulation in the cell plate was not reduced when cells were treated with CIPC or ES7. Both PIN1 and PIP2A, two plasma membrane localized transport proteins, accumulated in the cell plate.

The protein RAB1A is involved in vesicle tethering in both plants and mammals. In metazoans, the TRAPPIII complex activates RAB1 involved in ER-Golgi traffic and autophagy (Yang et al., 2016; Galindo and Munro, 2022). During mitosis in mammals, RAB1A localizes at distal regions of mitotic spindles (Marie et al., 2012). In Arabidopsis, RAB1A (AtRABD2a) plays a role in autophagosome formation in addition to promoting polarized growth (Zeng et al., 2021). AtRABD2a localizes to the Golgi and TGN/early endosomes, unlike the mammalian counterparts (Pinheiro et al., 2009). While most of the RAB-GTPAses in this study accumulated in the cell plate, RAB2A did not accumulate strongly in the cell plate, localizing more prominently in motile particles that may be Golgi bodies. These results are similar to that observed in Arabidopsis, where it co-localizes with a Golgi marker, and consistent with Golgi accumulation in maize biochemical fractionation experiments (Chow et al., 2008; Okekeogbu et al., 2019). In addition, in contrast to the other RABs or KNOLLE, RAB2A particle movements were faster and further increased during late stages of mitosis. In the future, it will be interesting to determine if this reflects Golgi movements during mitosis and cytokinesis.

The tubulin-binding herbicide CIPC generates split or multiple phragmoplasts which produce split or multiple cell plates (Clayton and Lloyd, 1984; Doonan et al., 1985; Eleftheriou and Bekiari, 2000; Young and Lewandowski, 2000). In our experimental conditions, CIPC treatment did not alter the recruitment of YFP-KNOLLE or RAB11D-YFP to the cell plate suggesting their localization may be independent of microtubule function or dynamics. However, CIPC caused significant defects in cell plate morphology detected by examining callose accumulation. In the future, it may be interesting to assess how microtubule dynamics are altered in CIPC treated cells.

Endosidin7 (ES7) is a chemical that inhibits cytokinetic specific callose deposition and cell plate maturation, often visualized as cell plate gaps. As a result, ES7 treatment disrupts the cell plate localization of KNOLLE and the RAB GTPase RABA2a during late cell stages of cell plate development (Park et al., 2014; Jawaid et al., 2022). Of the 12 callose synthases in Arabidopsis, CALLOSE SYNTHASE1/GLUCAN SYNTHASE-LIKE6 (CalS1/GSL6), CalS10/GSL8, and GSL10 are likely involved in cytokinesis (Záveská Drábková and Honys, 2017). In Arabidopsis, CalSI forms a complex with UDP-glucose transferase and localizes to the cell plate when expressed in tobacco cells (Hong et al., 2001). However, *cals1* mutants do not have defects in cell plate formation, while *gsl8* and *gls10* mutants have both reduced callose accumulation and defects in cytokinesis (Chen et al., 2009; Saatian et al., 2018). Although ES7 has a profound effect on cytokinesis in Arabidopsis and algae (Park et al., 2014; Davis et al., 2020), ES7 treated maize cells did not have defects in callose accumulation, cell plate morphology or altered YFP-KNOLLE or RAB11D-YFP accumulation. While earlier studies suggest that ES7 indirectly inhibits callose deposition (Park et al., 2014), the target of ES7 is still unknown. Whether lack of cell plate and root growth defects in maize roots and leaves are a result of poor ES7 uptake, lower affinity binding or other reasons is also unknown. Future experiments will clarify whether or not maize contains the ES7 target or whether it is sensitive to higher ES7 concentrations in germination treatments.

As demonstrated here, cell division in maize epidermal cells is a dynamic process that can be visualized at high spatial and temporal resolution. These markers highlight both conserved and potentially unique roles of proteins involved in cell division across the diversity of plants. Further experimental and functional studies using these markers will help clarify the role of these proteins and the spatial and temporal control of maize cell divisions.

## Data availability statement

The original contributions presented in the study are included in the article/**Supplementary Material**. Further inquiries can be directed to the corresponding author.

## Author contributions

LA, MB, VH, MD, AC, AR, BV, SS, AU, SM, CR designed and performed experiments, prepared the figures, and wrote the draft. GD provided ES7 and edited the manuscript, RS took images for ES7 experiments in Arabidopsis. GS, BD, DBV, and AN contributed to interphase microtubule measurements. CR conceptualized and designed the research, provided supervision, acquired funding and wrote the final manuscript. HS, XZ, and AS designed and generated fluorescent protein constructs EB1-mCherry and YFP-KNOLLE. All authors contributed to the article and approved the submitted version.
