# Supplementary material for: Subcellular positioning during cell division and cell plate formation in maize
Source: Front Plant Sci. 2023 Jul 7;14:1204889. doi: 10.3389/fpls.2023.1204889 (PMC10360171; doi:10.3389/fpls.2023.1204889)
Supplement: Supplementary file 1 [file DataSheet_1.docx]

| **Fluorescent protein** | **Forward Primer** | **Reverse Primer** | **Annealing temp** | **Other conditions** | **Extension time** | **Expected band size** |
| --- | --- | --- | --- | --- | --- | --- |
| EB1-mCherry | CTT GTG AGG GTG GCA TTG GAT TC | CCAGAGGAACCATACAGGGACAG | 58C | KOD w/DMSO | 1 min. | 915bp WT, 1.7kb EB1-mCherry |
| YFP-TUBULIN | GCAAGGTTTCGATTTCCGTA | GGTTTCGGGTGATCCCTATT | 53C | MyTaq no DMSO | 1 min. | 734bp WT, 1.5kb YFP-TUBULIN |
| CFP-TUBULIN | GCGACGTAAACGGCCACAAGTTCAG | CGGAAGCAGATGTCGTAGAGC | 59C | KOD w/DMSO | 2 min. | 1.3kb CFP-TUBULIN |
| RANGAP-YFP | N/A |  |  |  |  |  |
| HISTONE1.1-YFP | AGCCCAAGTCCAAGACCGCTGTG | CTGAACTTGTGGCCGTTTACGTCGC | 62C | MyTaq no DMSO | 45sec. | 529bp HISTONE1.1-YFP |
| YFP-KNOLLE | GCCAGACCGTCTTCAAATCATGC | CTGAACTTGTGGCCGTTTACGTCGC | 57C | MyTaq no DMSO | 45sec. | 711bp YFP-KNOLLE |
| GLOSSY-mRFP | TCTCCATTCTTCCCGAGTCC | TAGCCGAGTTGGATGCCTTT | 56C | MyTaq no DMSO | 1min. | 600bp WT, 1.4kb GLOSSY-mRFP |
| PDI1-YFP | GCGATAACCACAGGGACCCAAC | CACTCCATACCCACAGCCTAATC | 57C | MyTaq no DMSO | 1min. | 500bp WT, 1.2kb PDI1-YFP |
| PIP2A-CFP | TCGTCTGCCTCCAGTTCTATTCG | CTGAACTTGTGGCCGTTTACGTCGC | 58C | MyTaq no DMSO | 45sec. | 800bp PIP2A-CFP |
| PIN1-YFP | CATCACGCTCTTCTCCCTGTC | TACTTGTTCGCCTTGCCCTGC | 57C | MyTaq no DMSO | 1min. | 600bp WT, 1.3kb PIN1-YFP |
| RAB1A-CFP | AAG CAT CTC CAT CCT TCG CTC C | GCA AAC ATT CCA AGA GGC ACC | 57C | MyTaq no DMSO | 1min. | 470bp WT, 900bp RAB1A-CFP |
| RAB2A-YFP | N/A |  |  |  |  |  |
| RAB11D-YFP | GTGCCTGCTTTCCATCACC | CTTGTACAGCTCGTCCATGC | 56C | MyTaq no DMSO | 1min. | 500bp WT, 1kb RAB11D-YFP |

**Supplementary Table 1:** List of primers used in this study
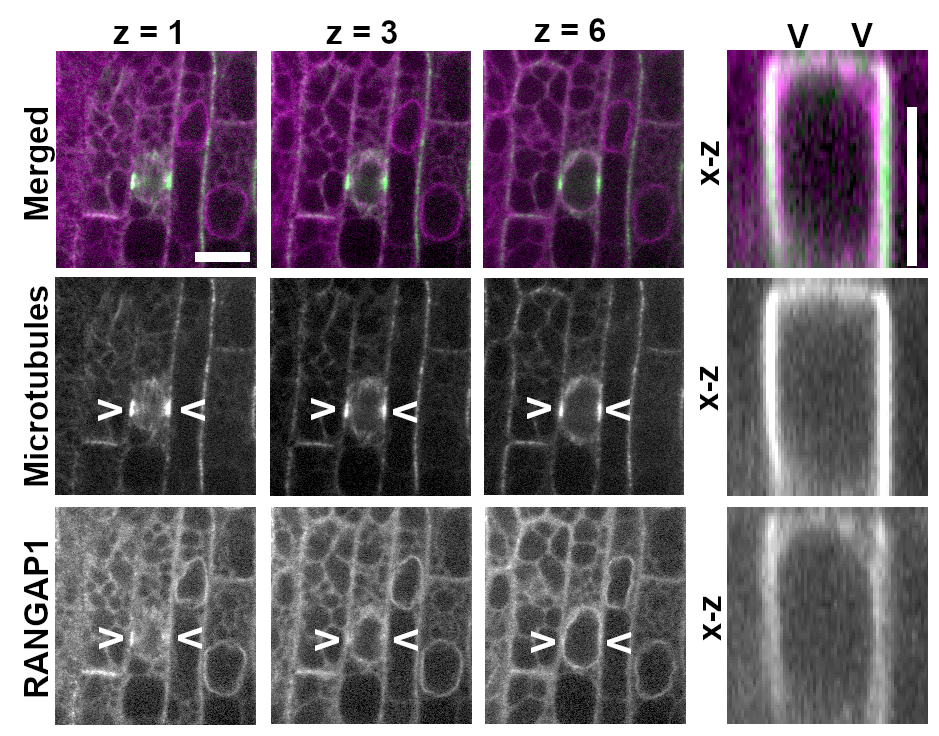


**Supplementary Figure 1: RANGAP accumulates in a slightly different location than the PPB.** RANGAP1-YFP is false colored magenta, while microtubules indicated with CFP-TUBULIN are false colored green. The left panels are a few Z stacks, with 0.5 µm interval. On the right is an x-z projection (scaled at 300%). Note that the magenta along the sides represents the nuclear envelope, which is clearly distinct from the PPB, while the very top of the x-z projection highlights the most obvious separation between RANGAP1 and microtubules at the top of the cell, indicated with arrowheads. Bars are 10 µm.


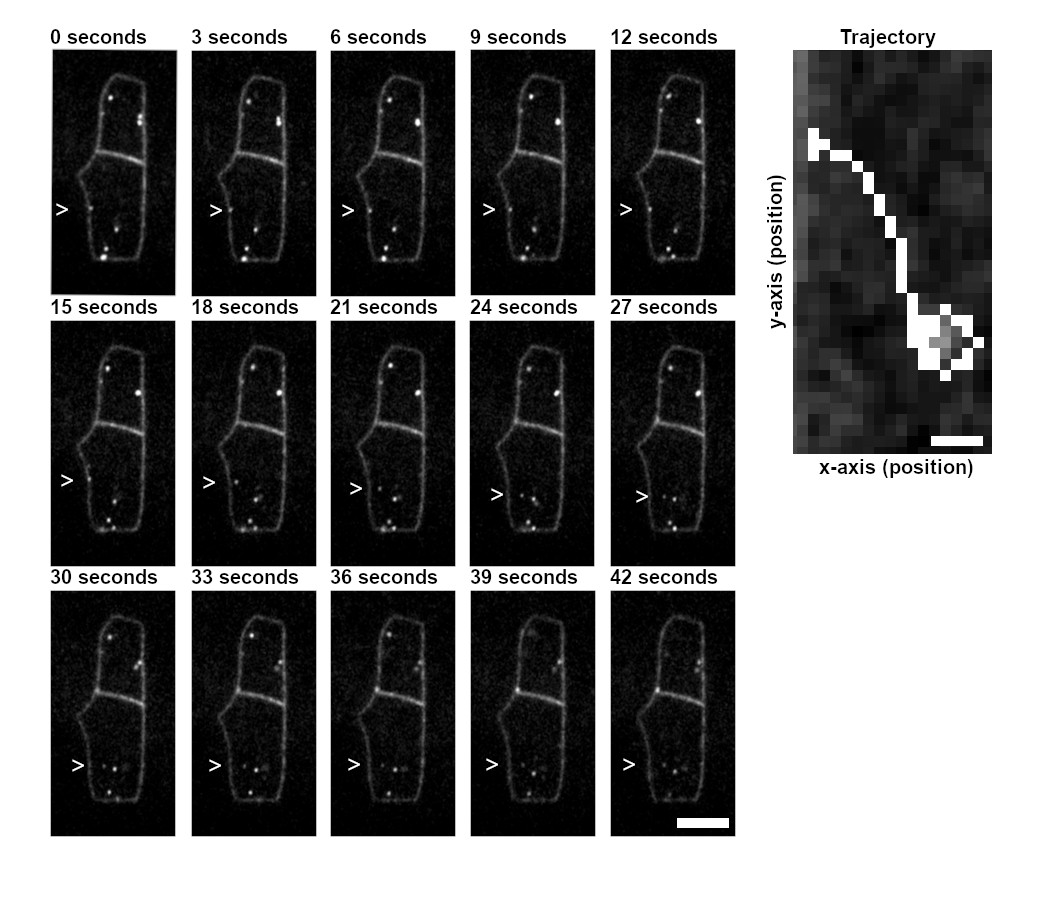


**Supplementary Figure 2: YFP-KNOLLE particle tracking and movement**. Arrowheads track the particle throughout the timelapse, scale bar 10 µm. Particle is stable at first and then moves down in the y-axis and towards the middle of the cell in the x-axis. Trajectory output of FIJI plugin Mosaic, Trajectory panel of figure (right), scale bar 1 µm. The particle coordinates were exported onto Excel or Google Sheets and converted from pixels to microns. The slope of the particle movement between frames was calculated using the slope formula m = (y_2_-y_1_)/(x_2_-x_1_) before taking the absolute value of all the numbers. The values were then divided by the interval of the time lapse (in seconds) to account for the time elapsed between each frame. Afterwards, the speeds were averaged to generate an average speed of that particle.


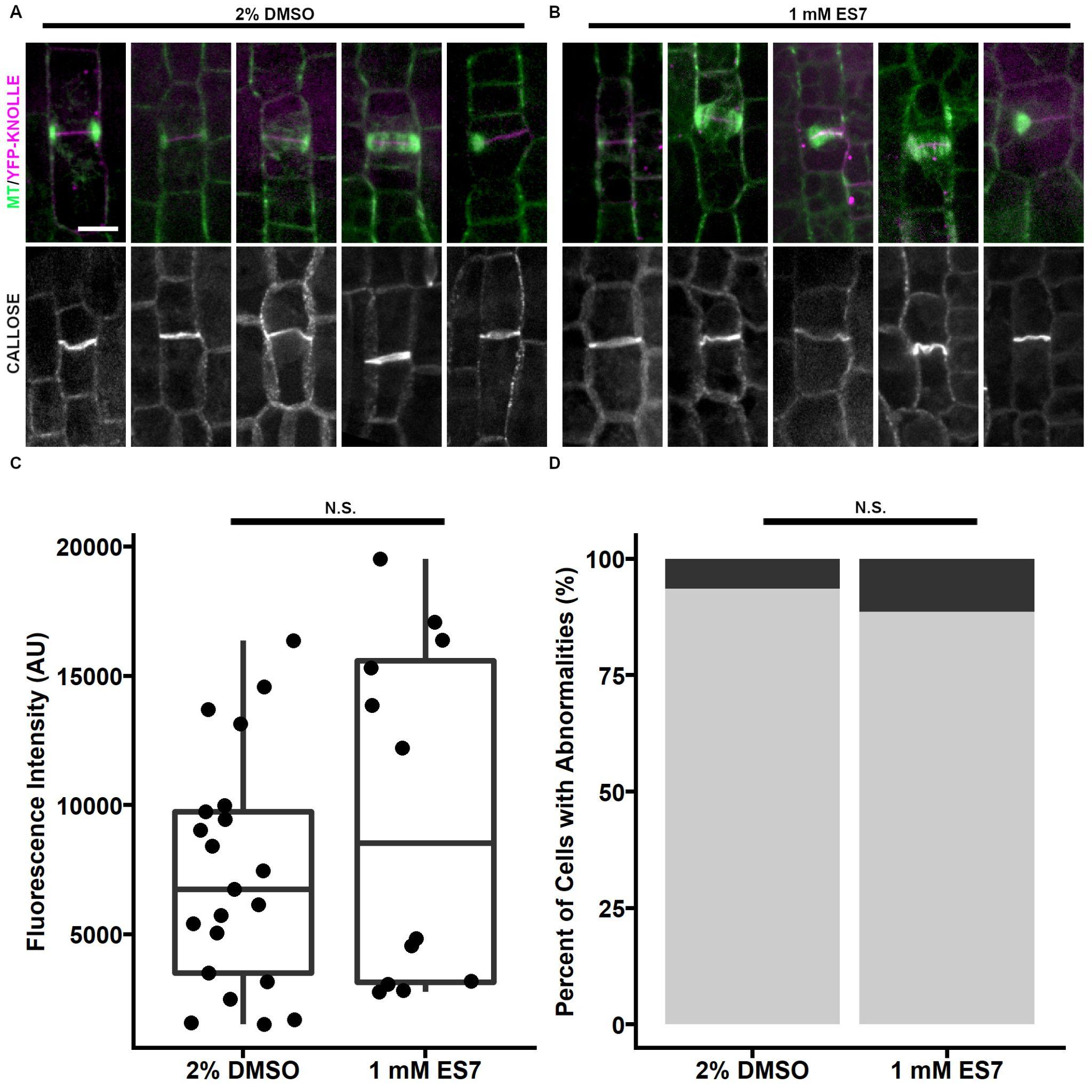


**Supplementary Figure 3: ES7 treatment does not disrupt localization of cell-plate specific YFP-KNOLLE or alter callose accumulation at cell plates.** A-B) YFP-KNOLLE (magenta) and microtubules (CFP-TUBULIN, green) are shown in the first row from the abaxial side of maize leaves in regions with symmetrically dividing cells. Callose staining was performed after ~3 hours of 2% DMSO (A) or 1mM ES7 (B) treatment in the second row. C) Box plot of fluorescence intensity between treatments. 2% DMSO average is 7379 ± 4451 AU, 1mM ES7 average is 9635 ± 6621 AU. Not significant by Welch’s two sample t-test, p-value = 0.31. 2% DMSO treated plants (n = 3 plants, n = 21 cells) and 1 mM ES7 treated plants (n = 3 plants, n = 12 cells) For each dot, 5 measurements were made of fluorescence intensities within the image (background) or within the cell plate and averaged. Background intensities were subtracted from cell plate intensities to generate the arbitrary fluorescence measurements for these graphs. D) Abnormalities, such as cell wall stubs and split cell walls, were detected in 2% DMSO (6.38%, dark grey bars, 9 cells of total 141 cells) and in 1mM ES7 (11.42%, dark grey bars, 20 of total 175 cells). Normal callose accumulation is represented by light grey bars. Plants used for each treatment = 3. Cells used for each treatment to measure fluorescence: 1mM Control = 21, 1mM ES7 = 12. N.S. not significantly different via Fisher’s exact test p-value > 0.05.

**
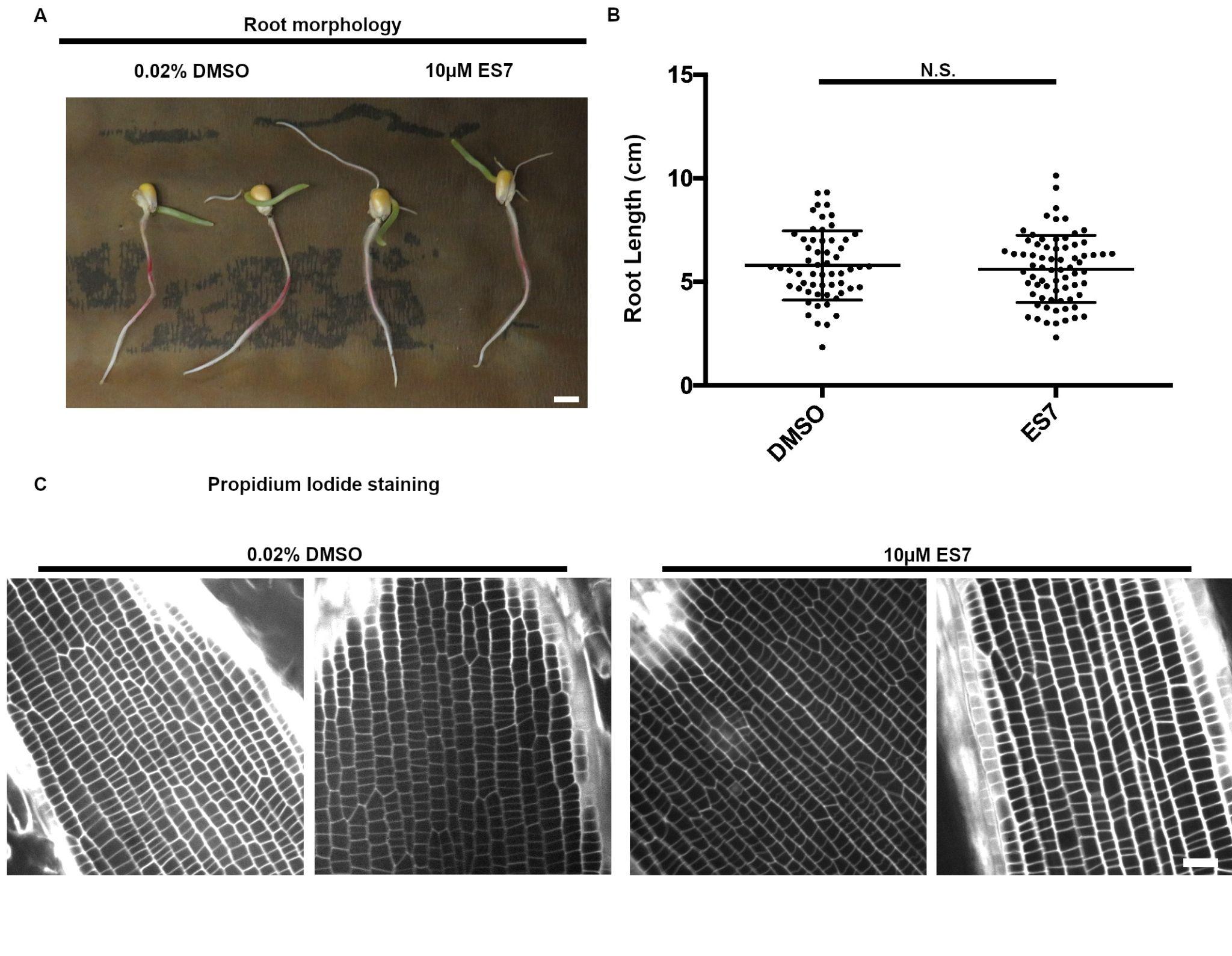
**

**Supplementary Figure 4: Treatment with 10 µM ES7 for 5 days does not affect root growth or generate cell wall stubs in maize seedlings.** A) Root scans of 5-day old seedlings grown in DI water and 0.02% DMSO (left) and 10 µM ES7 (right). Scale bar is 1 cm. B) Root length (cm) of 0.02% DMSO (3 replicates, 58 plants) vs 10µM ES7 treated plants (3 replicates, 71 plants). Average of 0.02% DMSO = 5.8 cm ± 1.7, ES7 = 5.6 cm ± 1.6. No significant differences detected with Mann-Whitney *U* test, p value > 0.1. C) PI staining of 0.02% DMSO and 10 µM ES7 treated plants in secondary root tips. 3 replicates total (ns between replicates), 40 plants were examined for each treatment. Scale bar is 30µm.

**
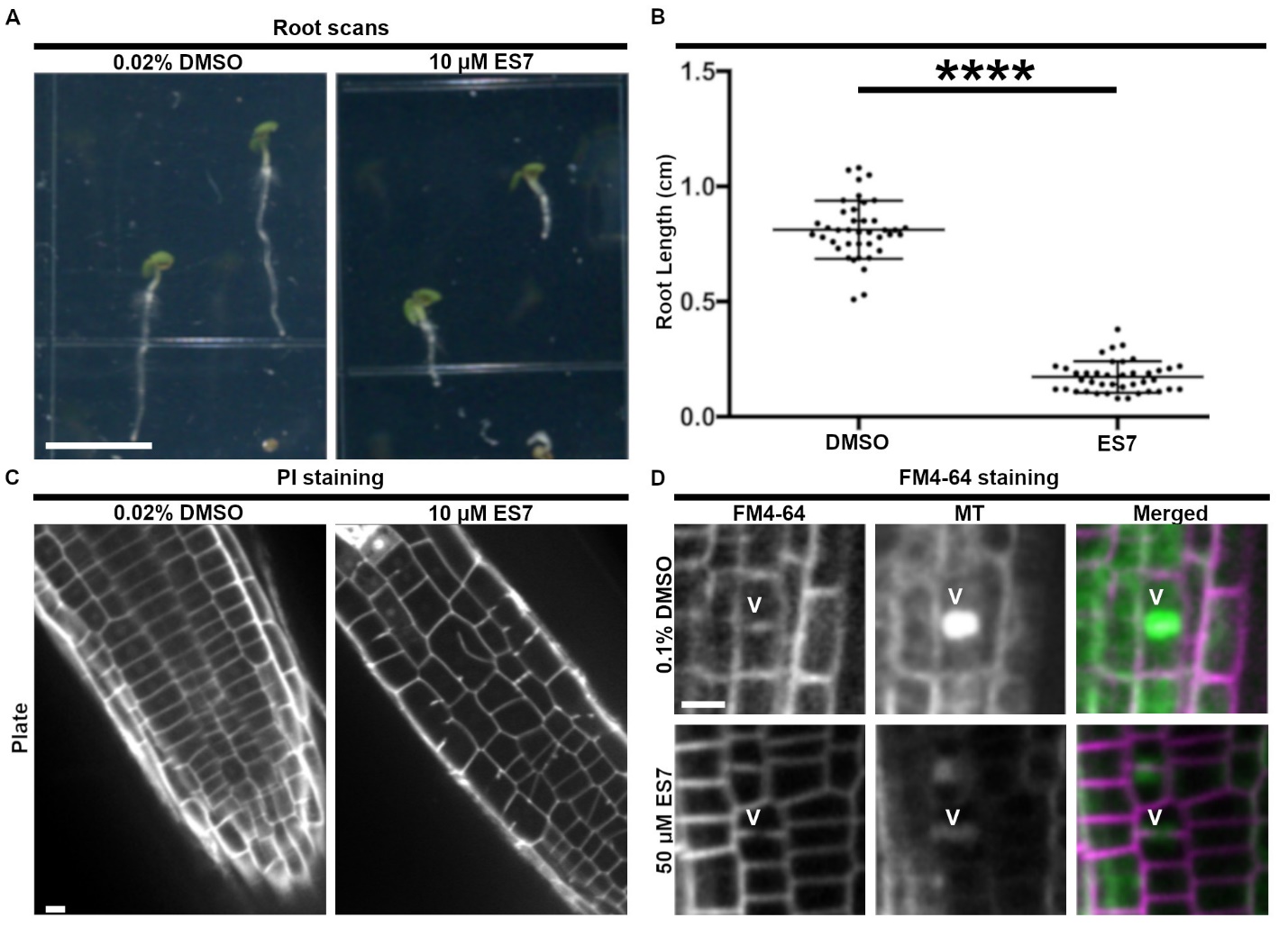
 Supplementary Figure 5: ES7 treatment reduces root length and generates cell wall stubs and cell plate defects in Arabidopsis seedlings.** A) Scans of 5-day old seedlings grown in ¼ MS media with 0.02% DMSO (left) and 10 µM ES7 (right). Scale bar is 0.5 cm. B) Root length (cm) of 0.02% DMSO and 10µM ES7 treated 5-day old seedlings. 2 replicates total (ns between replicates), 40 plants each treatment measured. Average of 0.02% DMSO = 0.81cm ± 0.13, ES7 = 0.18cm ± 0.08. Mann-Whitney *U* test p value <0.001 ****. C) Propidium Iodide (PI) staining of root tips in seedlings grown for 5 days on ¼ MS plates with 0.02% DMSO and 10 µM ES7 Scale bar is 10 µm. D) Wild-type seedlings with CFP-TUBULIN pulse-treated with 0.1% DMSO (arrowhead shows a normal cell plate) and 50 µM ES7 (arrowhead shows a disrupted cell plate) stained with FM4-64 (2 µM). In the merged image CFP-TUBULIN labeled microtubules are green and FM4-64 labeled membranes are magenta. Scale bar is 10 µm, all panels in D are the same magnification. 50 µM ES7 images are a maximum projection of 2 frames, z-step = 1µm.


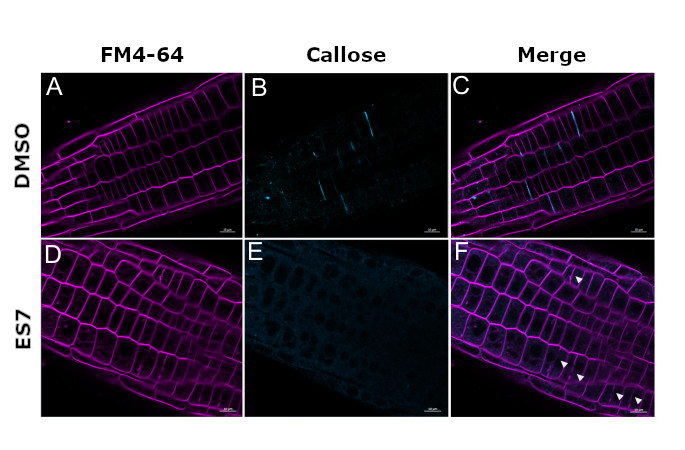


**Supplementary Figure 6: Membrane staining and callose deposition of Arabidopsis root cells treated with 50 µM endosidin 7 (ES7) or 0.1% DMSO.** A-C). Control plants treated with 0.1% DMSO. A) 2 µM FM4-64 plasma membrane staining. B) Aniline blue staining of late-stage cell plates in dividing cells. C) Overlay of the two channels is shown. D-F). 50 µM ES7 treatment (2 hours) leads to cell plate defects shown by 2 µM FM4-64 staining (D, E, F arrowheads). C, F) In contrast to the control, there is no detectable callose deposition in arrested cell plates. Scale bar is 10 µm. Images are representative of 10 seedlings per treatment.
